# “Good job!”: Therapists' encouragement, affirmation, and personal address in internet-based cognitive behavior therapy for adolescents with depression

**DOI:** 10.1016/j.invent.2022.100592

**Published:** 2022-11-15

**Authors:** Ida Berg, Vera Hovne, Per Carlbring, Claudia Bernhard-Oettel, Martin Oscarsson, Jakob Mechler, Karin Lindqvist, Naira Topooco, Gerhard Andersson, Björn Philips

**Affiliations:** a Department of Psychology, Stockholm University, Stockholm, Sweden; b Department of Psychology, Uppsala University, Uppsala, Sweden; c Department of Behavioural Sciences and Learning, Linköping University, Linköping, Sweden; d Department of Biomedical and Clinical Sciences, Linköping University, Linköping, Sweden; e Center for Psychiatry Research, Department of Clinical Neuroscience, Karolinska Institutet and Stockholm Health Care Services, Stockholm County Council, Stockholm, Sweden

**Keywords:** Therapist behavior, Internet cognitive behavior therapy, Depression, Patient adherence, Adolescent

## Abstract

Internet-delivered interventions are generally effective for psychological problems. While the presence of a clinician guiding the client via text messages typically leads to better outcomes, the characteristics of what constitutes high-quality communication are less well investigated. This study aimed to identify how an internet therapist most effectively communicates with clients in internet-delivered cognitive behavioral therapy (ICBT). Using data from a treatment study of depressed adolescents with a focus on participants who had a positive outcome, messages from therapists were analyzed using thematic analysis. The study focused on the therapist's 1) encouragement and 2) affirmation, and how the therapists used 3) personal address. The analysis resulted in a total of twelve themes (Persistence Wins, You Are a Superhero, You Make Your Luck, You Understand, Hard Times, You Are Like Others, My View on the Matter, Time for a Change, Welcome In, Let Me Help You, You Affect Me, and I Am Human). Overall, the themes form patterns where treatment is described as hard work that requires a motivated client who is encouraged by the therapist. The findings are discussed based on the cognitive behavioral theoretical foundation of the treatment, prior research on therapist behaviors, and the fact that the treatment is provided over the internet.

## Highlights

• Characteristics of what constitutes high-quality communication are largely unknown.
• We identified how an effective internet therapist communicates with depressed clients.
• Therapists' encouragement, affirmation, and personal address were in focus.

## 1 Introduction

Internet-based psychological treatments have been provided since the mid-1990s and are developing rapidly (Smoktunowicz et al., 2020). Most studied treatments are forms of internet-based cognitive behavioral therapy (ICBT; Andersson et al., 2019, Andersson et al., 2019). Increased accessibility, opportunities for clinical innovation, and cost-effectiveness are arguments in favor of these interventions (Donker et al., 2015). ICBT is now beginning to be implemented in healthcare worldwide (Titov et al., 2018).

Research has shown that ICBT effectively treats various psychological problems and mental disorders (Andersson et al., 2019a). Guided ICBT is more effective than wait-list controls and appears as effective as face-to-face treatment (Carlbring et al., 2018). Additionally, the effects appear to be long lasting (Andersson et al., 2018).

While there are different types of ICBT, one common characteristic is that the treatment material is delivered and administered on a digital platform. Treatments are generally based on standardized self-help material for clients, such as texts and images/videos. Support from therapists may be offered, usually referred to as *guided* ICBT. The treatment platforms typically have technical solutions for asynchronous communication between clients and therapists, such as messaging functions (Andersson and Carlbring, 2022).

Communication and therapist support in ICBT varies, but effects are generally greater in guided ICBT than unguided ICBT (Furukawa et al., 2021). Therapist support is associated with positive effects on symptom reduction, adherence to treatment, and dropout rates. What constitutes good therapist support is still unclear. While one review found evidence for a strong correlation between the degree of support and outcome in the treatment of depression, where a higher degree of support included contact before and/or after treatment (Johansson and Andersson, 2012), few studies have examined the actual dose-response relationship (Baumeister et al., 2014). It has been debated how therapist experience relates to the effectiveness of the support. For example, therapist support from inexperienced therapists seems as effective as that of experienced ones (Andersson et al., 2012). Likewise, support of a more practical and technical nature, without clinical advice, may be comparable to support from a clinician (Titov et al., 2010). It is also undetermined who benefits most from therapist support. The need may for example be greater among adolescents than among adults. For example, a study on ICBT for adolescents with depression and anxiety showed that those receiving support from teachers completed treatment exercises to a greater extent than those undertaking treatment on their own (Neil et al., 2009).

So far, research on internet-based psychological treatment has been mainly quantitative, with relatively few published qualitative studies (Patel et al., 2020), thus quantifying associations but overlooking participants' or therapists' experiences. These, however, would give valuable insights concerning potential differences, advantages, challenges, or hindrances when therapy is given via a platform and not face-to-face. Notable exceptions include a study by Beukes et al. (2018), on participants' experiences of ICBT for tinnitus, and Berg et al. (2020), on what adolescents learn and recall from ICBT for depression. In another qualitative study on ICBT for adults with depression, Bendelin et al. (2011) found that participants wanted support from their therapists, however too much contact with the therapist could cause participants to doubt their ability and autonomy, both considered crucial in successful treatment. Thus, the authors conclude that much remains to be explored in terms of how therapist support in ICBT should be given to clients.

### 1.1 Therapist behavior and communication

Taking a closer look at what therapists are doing in ICBT, Sánchez-Ortiz et al. (2011) coded therapist messages in a randomized trial of ICBT for adults with bulimia. Messages were coded into three broad categories: supportive comments (encouraging or generally affirming), CBT comments (references to specific thoughts or feelings, with suggestions of action), and technical or study-related comments (e.g., regarding the treatment platform). The authors found that almost all messages, 95.4 %, contained at least one supportive comment, while CBT comments were found in 14.7 % of messages and technical or study-related comments in 13.6 % of messages. The fact that supportive comments far outnumber CBT comments is discussed as surprising. The authors consider whether the frequent supportive comments are an attempt to build an alliance, in lack of face-to-face contact, and whether the client messages may lack enough details for therapists to suggest tailored CBT strategies. The authors further consider that the main role of the therapists is to aid the engagement with and assimilation of the CBT material, which perhaps is not best achieved with CBT techniques, and that some CBT techniques, such as Socratic questioning, may be particularly difficult to use in the context of infrequent online text messaging. From another perspective, a high proportion of supportive relative to CBT comments could be expected since the communication should not replicate what is covered in the modules, but instead be encouraging, and in some cases clarifying.

Therapist behavior in ICBT for adults with generalized anxiety disorder has been studied by Paxling et al. (2013). Eight distinct therapist behaviors were identified, with task reinforcement (40 %), self-efficacy shaping (34 %), and task prompting (12 %) being the most common. Task reinforcement had a positive association with symptom improvement. Both task reinforcement and self-efficacy shaping would be coded as supporting comments according to the definition of Sánchez-Ortiz et al. (2011). Thus, encouraging the client and cheering them on, without a clear connection to treatment content, was common in both studies.

In a study of therapist behavior in ICBT for adults with depression (Holländare et al., 2016), nine distinct behaviors were identified. The results were unanimous with the previously mentioned studies: general cheering, in the broad sense, accounted for a large part of the communication, while comments, specific advice, and explanations with a clear association to CBT accounted for a smaller part. The most common behavior was encouraging (31.5 %), affirming (25.1 %), and guiding (22.2 %). Both affirming and encouraging correlated with symptom improvement.

Schneider et al. (2016) also examined therapist behavior in ICBT for adults with depression, proceeding from the same behaviors described by Paxling et al. Three additional behaviors were identified. The most common behaviors were alliance bolstering (21 %), administration (16 %), task reinforcement (14 %), and task prompting (14 %). No correlation was found between any therapist behavior and symptom improvement. However, there was a correlation between other behaviors, including administrative statements and task prompting, and lesser symptom improvement. One possible explanation suggested by the authors was that therapists may have increased some behavior in communication with clients demonstrating increased symptom severity.

In summary, research on therapist behaviors in ICBT is scarce and inconsistent. Treatment studies focus on different conditions and differ in design. The type of therapist support varies, as does the selection criteria. There is no broad consensus on how different behaviors shall be defined and distinguished from one another, making comparisons even more difficult. The frequency of different behaviors differs between studies, even studies using the same categorization. However, a weak pattern emerges: affirmation, encouragement, and other non-CBT-specific behavior are more prominent than behavior with a clear connection to the treatment material. While there is no coherent image of therapist behavior associated with treatment outcome, reinforcing upcoming tasks, or encouraging, does seem to correlate with symptom improvement (Holländare et al., 2016; Paxling et al., 2013).

With previous research on therapist behavior in ICBT being predominantly on adults and quantitative, the purpose of this study was to qualitatively explore therapists' encouragement, affirmation, and personal address in communication with adolescent clients in ICBT for depression, using thematic analysis. A better understanding could inform about possible developmental considerations and whether therapist support may be more important for adolescents than adults.

Addressing this void, the overall aim of this study was to increase awareness of how therapists can provide CBT via the internet, thus providing knowledge that can ultimately be of practical use to therapists. By means of qualitative exploration, we investigated therapists' written communication with adolescent clients in ICBT for depression, to better understand therapist behavior with respect to encouragement, affirmation, and personal address signaled and verbalized when practicing ICBT.

## 2 Method

### 2.1 Context of the study

Data for this study came from the ERiCA (Early Internet-Based Interventions for Children and Adolescents) project at Stockholm University, in collaboration with Linköping University, comparing ICBT against internet-based psychodynamic therapy (IPDT) for adolescents with depression (Mechler et al., 2022). The ICBT intervention consists of eight modules over ten weeks. Therapist support consists of longer, weekly messages, and a 30-minute text-based chat once a week. The treatment is developed from an ICBT intervention for adults with depression (Topooco et al., 2018), which, in turn, is based on face-to-face treatment protocols, focused on behavioral activation and cognitive restructuring (Andersson et al., 2005). The ICBT intervention has previously been evaluated in two randomized controlled trials, showing lasting effects on symptoms of depression (Topooco et al., 2018, Topooco et al., 2019).

### 2.2 Participants

Therapists were recruited from the psychology programs at Stockholm University, Uppsala University, and Karolinska Institutet (Sweden). In total, 40 therapists worked in the study, of which 18 provided the ICBT intervention. These were all students in their final semester, having completed basic psychotherapy training in CBT, and having prior clinical experience. All therapists received a full day of specific training for the project and had continuous supervision by a licensed CBT psychologist.

### 2.3 Messages

The therapists were instructed to provide clients with individual feedback on completed exercises during the course of treatments. They had access to examples of what such communication could entail under different phases of treatment. If the clients had not completed their exercises, the therapists would still send a weekly message about the treatment. Composing the messages was not to take longer than 15 min. In addition to the longer messages, therapists were also allowed to send shorter messages, two to three sentences long, in response to clients' questions and exercises. As long as they kept within the scope of the treatment, therapists were free to formulate their messages as they saw fit, using a tone they felt comfortable with. The treatment also included weekly chat sessions between therapists and participants; this communication was not analyzed in the present study. To differentiate communication in messages versus sessions, therapists were instructed not to encourage excessive back-and-forth messaging in their feedback messages. For example, a practical application of this guideline involved therapists avoiding sending follow-up questions to the client in their feedback messages on completed exercises.

The ERiCA project received ethics approval from the Swedish Ethical Review Authority (ref. number: 2019-03023), with both clients and therapists providing informed consent. All data used for this study was pseudonymous. Quotes containing identifying information were excluded.

### 2.4 Sampling

From a pool of ICBT treatments where clients had been actively participating (i.e., completing exercises almost every week) and experienced a reduction in depressive symptoms (i.e., a reduction of ≥6 points on the Quick Inventory of Depressive Symptomatology [QIDS-A17-SR]), five full treatments were randomly selected by an independent person using a true random number service (RANDOM.ORG). Additional treatments could have been included, but data had reached saturation. From these treatments, all text-based communication between clients and therapists was extracted and compiled, amounting to 19,779 words in total. After excluding messages from clients and administrative messages (e.g., regarding scheduling) 49 messages, amounting to 13,803 words in total, remained (range 1650–4528 words per treatment).

### 2.5 Analysis

Thematic analysis, as described by Braun and Clarke, 2006, Braun and Clarke, 2013, was used to explore therapists' encouragement, affirmation, and personal address in messages to their clients. Both coders (the first and second author) were final-semester psychology students, both having worked as therapists in the ERiCA project, however in later cohorts than those included in sampling for this paper. The analytic work was discussed, refined, and finally reported on in close cooperation with two senior researchers with expert knowledge about internet interventions (PC) and qualitative research methods (CBO).

The first step was a deductive analysis, identifying the therapist behavior 1) encouragement, 2) affirmation, and 3) personal address in the messages. Encouragement was defined as any statement to encourage desirable client behavior, including reinforcing previous behavior and prompting future behavior. Affirmation included noticing, acknowledging, and expressing interest in the client's thoughts, feelings, and actions, e.g., validating, interpreting, normalizing, and summarizing. Personal address involved self-disclosure, e.g., therapists referring to themselves, using themselves as a natural reinforcer, or otherwise making themselves visible. See the Appendix for the coding template, including example statements.

Departing from these specific therapist behaviors and their definition, the first and second authors first coded one treatment separately. Overall, the consensus was strong (72 % interrater code agreement). Disagreements and uncertainties were discussed between the coders, further clarifying the definitions and delimitations in the coding template. The remaining material was divided and coded separately by both authors. The results were discussed between the coders and any remaining ambiguities were reviewed until a consensus was reached. The deductive coding resulted in a data set with 68 % of original data, including 158 statements of encouragement and 86 statements of affirmation and personal address, respectively.

The second part of the analysis was done jointly with the remaining data and followed the principles of inductive thematic analysis as outlined by Braun and Clarke, 2006, Braun and Clarke, 2013. Encouragement, affirmation, and personal address served as overall categories, and for one category at a time, all statements were worked through and individual codes were generated from data. Codes in each category were reviewed and clustered based on common patterns. Based on these, preliminary themes were formulated. This process included going back and forth between codes, themes, and data, with an ongoing conversation, joint reflection, and decisions by consensus. Finally, themes were defined, named, and described based on their connections.

## 3 Results

**Fig. 1:**
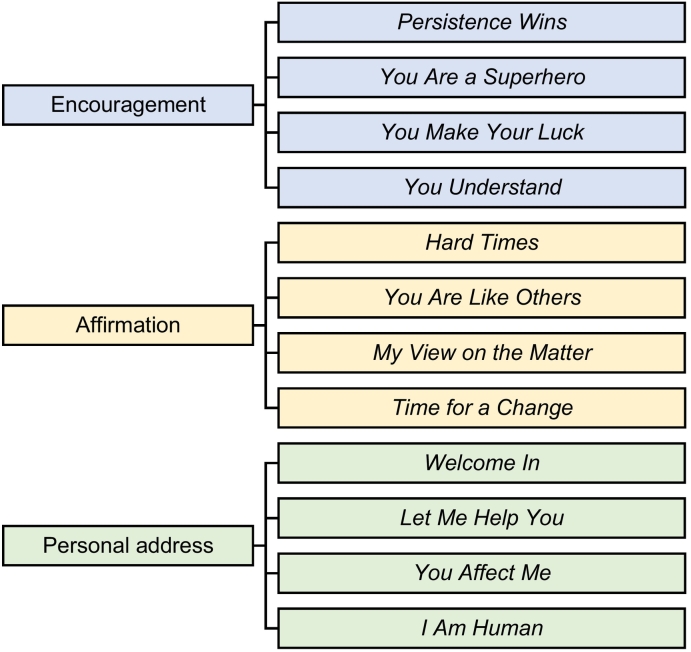
The thematic analysis results with themes ordered by therapist behavior.

The thematic analysis resulted in a total of twelve themes, four each in encouragement, affirmation, and personal address (see Fig. 1).

### 3.1 Encouragement

In Encouragement, four themes were identified: Persistence Wins, You Are a Superhero, You Make Your Luck, and You Understand. Persistence Wins is the most comprehensive and central theme of the category.

#### 3.1.1 Persistence Wins

A comprehensive theme in the therapists' encouragement involves describing the treatment as hard work, which the client needs to take on with great perseverance. The therapists encourage clients by stressing that they are doing a good job, that small steps count, and that it is important to continue working with the treatment, even when it feels difficult or when results are lacking.

A common way for therapists to encourage clients is with statements such as “Good job!” and “You are doing great work!” when the client has completed some element in the treatment, for example, completed an exercise, logged their activities, or dared to subject themselves to an anxiety-inducing situation.

Another important part of the therapists' encouragement is emphasizing the value of tackling hard work in small steps. This emerges as therapists stress that everything the clients do in treatment leads forward, and even the smallest effort counts. Every step is an important part of the larger, hard work clients must do to achieve the goal of feeling better. With this, therapists make it clear to clients that they are (or should be) active in treatment and that this activity, however small, is vital, as change occurs gradually and one thing builds on another. In line with this, therapists often direct encouragement toward clients starting activities, such as exercises, rather than toward the results.

*It's great that you have started on week 4! It looks like you've planned just enough of the moderately challenging activities and that's great :) just like with physical exercise, you shouldn't go all in at once, but start out small and build on that! Just like you've done.*

(Therapist no. 2)

Another aspect of Persistence Wins is when therapists encourage clients to continue working with the treatment even though it is difficult. This ranges from encouraging clients to tell the therapist how they are feeling, even though it feels unusual, to encouraging clients to complete activities even though they are anxious or show up in the chat despite being nervous. In this type of encouragement, the therapists show an understanding that treatment takes a toll on the clients.

*Great job with the exercises! You have really managed to plan for and do activities, even though you were anxious about them.*

(T1)

#### 3.1.2 You Are a Superhero

Part of Encouragement is about highlighting certain characteristics of clients and painting a picture of clients as superheroes. In You are a Superhero, therapists emphasize that clients are brave and strong. “Awesome” and “a Fighter” are monikers that therapists use to describe clients when encouraging them. Superhero clients are competent and have what it takes to do the right thing.

*You really are an absolutely amazing person who is so strong and awesome!*

(T2)

Therapists highlight the superhero traits as qualifications for coping with the hard work of treatment, as well as the stresses of everyday life. Thus, You are a Superhero is linked to Persistence Wins. Clients have the inherent strength required to keep fighting.

*Lastly, I really want to stress that you signing up for the treatment was very strong of you, and it is a first important step towards feeling better.*

(T4)

Therapists explain that the qualities that superheroes possess will help clients through treatment. However, the strengths described are quite vague, without a clear connection to specific tasks. Clients will make it through treatment because they are awesome in general, not because they are good at planning or quick learners.

Persistence Wins is about what clients do or should do, while You Are a Superhero is all about who the clients are. From the start, the therapists know that the clients are superheroes. When the clients succeed, for example in exercises, that proves what the therapists had always known: the clients are strong, fantastic, and brave. The superhero qualities are necessary for the hard work, and the hard work is proof that the clients are superheroes.

*I really think you are brave and strong who tackled your anxiety hierarchy and also attempted such a high-level exposure straight away! :)*

(T5)

In Persistence Wins, clients are given the choice to work or not. The superhero, however, is ever-present. The superhero is a prerequisite for treatment and is asserted by it. The clients go into treatment as superheroes, continue to be superheroes, and leave treatment as superheroes.

#### 3.1.3 You Make Your Luck

You Make Your Luck is closely linked to Persistence Wins, in that it is based on the fact that treatment is hard work. You Make Your Luck emphasizes clients' responsibility in treatment, and by extension their future well-being. You Make Your Luck is more common at the end of treatments, but also occurs earlier, as evident below where the therapist highlights the client's choice:

*You chose to go for a walk and talk to a friend when you were at your worst, and that was really wise of you. It's those types of feel-good activities we need more of when we are feeling low.*

(T4)

The therapists further emphasize that hard work must continue in the future and responsibility for that lies with the client. The theme also includes the idea that clients may shoulder this responsibility because they have put in work in the treatment and thus have a new set of skills to face future challenges.

*The fact that you continue doing the exercises, run a check 1–2 times a month (as we talked about in the chat), and remember what you wrote last time (being sad / feeling down does not mean that you are depressed again) is a winning concept!*

(T5)

#### 3.1.4 You Understand

In this theme, therapists focus on clients' understanding. When clients have read the module, completed exercises, and/or talked to their therapist in the chat, therapists point out that the work leads to understanding and insight. On the one hand, therapists' encouragement applies to the clients' understanding of the material:

*I've also read your Situation, Thought, Feeling form and you've really grasped the concept.*

(T1)

On the other hand, the understanding also applies to the clients themselves:

*(…) because just as you write, it is often when, for example, you are alone and you start ruminating about something or worrying that you might also start to feel worse, so that was a great insight you had! :)*

(T5)

In many cases, understanding is attributed to an intrinsic value. In this context, the purpose of the exercises is for clients to understand something new, as understanding is desirable in itself, and worth highlighting by the therapists.

The therapists also emphasize the importance of understanding differently. In these cases, therapists attribute increased importance to understanding, presenting it as part of the path to change and, by extension, improved well-being.

*You also say that you know that feelings and thoughts that affect you may appear when you are about to do things and just knowing this is a big step in the right direction!*

(T2)

In Persistence Wins, therapists describe the treatment as consisting of many small steps. You Understand is one of the steps required for clients to feel better. The step of understanding is consistently highlighted and emphasized by therapists, thus emerging as a pattern. Understanding may be viewed not only as one small step along the way but rather as the only major step. Because of this, You Understand is considered a separate theme.

### 3.2 Affirmation

In affirmation, the analysis resulted in four themes: Hard Times, You Are Like Others, My View on the Matter, and Time for a Change.

#### 3.2.1 Hard Times

This theme covers part of the therapists' validation. The therapists affirm that they can tell the clients are in a stressful situation. In Hard Times, therapists acknowledge and clarify what the situation is like and what the client is going through. This includes descriptions of life being hard, tough, rough, and heavy. There have been hard times, these are hard times, and there may be hard times ahead.

*I see that you are having and have had a hard time in several different ways and that you are struggling with several difficult things.*

(T5)

There are also specific descriptions of circumstances that therapists highlight as problematic. These are circumstances that therapists link to the hard times. Circumstances include high demands, self-criticism, stress, as well as loneliness.

*You also think about your self-esteem in relation to achievements, where you also say they are linked. It is really hard to have to set such high standards for yourself (…)*

(T2)

#### 3.2.2 You Are Like Others

The therapist's affirmation is also about normalizing. For example, telling clients that they are like others. You are Like Others emphasizes normalcy. The clients are no different from others, they are the same. It becomes a way of telling clients they are not alone in their experiences.

The identity of “others” varies: therapists write that the clients are like other adolescents and/or others with depression. You Are Like Others is also about telling clients they think, feel, and react in ways that are reasonable and expected, given the situations they are in.

*The problems you describe having today, being unsure of who you are, ruminating, and experiencing anxiety are similar to the experiences of other adolescents who feel bad.*

(T2)

Emotions are a prominent topic in the theme. The therapists convey to clients that it is okay and natural to have emotions, that all emotions are allowed, and that feelings are not to be ashamed of.

*I understand that it weighs on you! Of course, just like all humans, you need to feel seen and affirmed in your emotions, and it must be hard to constantly keep them bottled up inside.*

(T5)

Part of the theme concerns depression. Therapists normalize clients' experiences by explaining that they are common in depression.

*You also wrote in the chat that your well-being and self-esteem are often linked; when you feel bad, you also feel inferior. That is often the case - when you get depressed, your thoughts about yourself often become much darker.*

(T3)

#### 3.2.3 My View on the Matter

In My view of the Matter, therapists affirm clients by framing their stories. The theme is different from Hard Times and You Are Like Others in that therapists, in addition to validating and normalizing clients' experiences, offer a way of understanding them. This may be described as therapists looking at clients' stories through their lenses and offering these lenses to the clients.

*I see that you have described a situation where you experience a form of adversity or failure that you feel you cannot control. In the moment, it feels better to worry about it and try out all sorts of ways of dealing with it, even if it takes time and energy from what is more important or fun for you. Maybe trying to control the situation reduces anxiety or other even more difficult feelings right then and there? In this way, your reaction and behaviour are perfectly reasonable. But, just as you were saying, the long-term consequence is that you have more anxiety and also don't have the energy to do things that you can control or things that make you feel better.*

(T4)

As illustrated in the example above, therapists often use the explanations found in the treatment modules, applying them to clients' situations. For example, therapists use words such as “situation”, “behavior”, “short- and long-term consequences” and “vicious circle”. Therapists may convey that insight is not enough to solve problems, that the clients' previous experiences impact what they are feeling today, that the clients' actions impact their well-being, and that one way of dealing with emotions is leaving them be.

Therapists may also use words that are more familiar or closer to the client's choice of words when extending their view on the matter:

*It seems like you need to do less sleeping, while doing more riding, hanging out with friends, and training the dog.*

(T3)

In My View on the Matter, some explanations are not clearly set out in the treatment modules:

*You also mention a very clear example of what happened to you in a situation where you did not express your feelings to your parents and then hurt yourself, and it sounds to me like this is a theme where you suppress your needs and feelings and they then manifest in, among other things, self-harm.*

(T1)

#### 3.2.4 Time for a Change

In affirmation, there is also a pattern where therapists write about future or past changes, depending on how far along they are in treatment. In the beginning, therapists affirm that it is time for a change. Clients do not have to keep feeling bad, change is possible, and now is a good time. The statements revolve, inter alia, around the fact that the clients have sought help. The fact that they have sought and started treatment is highlighted. It is a sign that they are now taking themselves seriously and that winds of change are blowing.

*At the same time, I am impressed that you have taken control of your health and sought help. It shows that you are driven and ready to make changes in your life, and I really think you can be proud of that.*

(T1)

This theme also includes an affirmation of change in the clients' well-being, usually for the better.

*According to your self-ratings, it looks like you are mostly feeling better, more so than feeling worse, which is great to see!*

(T4)

Toward the end of treatment, Time for a Change includes affirmation of changes that have occurred, that have made clients better prepared to face future difficulties.

*Based on your descriptions, it seems as if the treatment has helped you in several ways. It has become clear to you how your actions impact your well-being. (…) You have also discovered that you easily start worrying about what can go wrong in different situations, but by daring to expose yourself to the things you worry about, you have been given an opportunity to disprove those worrying thoughts. You've also learned that anxiety always wears off eventually, and that you can persevere even when you are at your worst.* (T4)

*You have now pulled yourself out of a depressed state once and should you experience periods of depression in the future, you have the important skills and knowledge to pull yourself out of it again.*

(T3)

This part of Time for a Change is similar to You Make Your Luck, in the category of Encouragement. What separates the two is that in Time for a Change, therapists affirm the situation at hand and what has happened within clients, without appraising clients' actions or telling clients what to do. In You Make Your Luck, therapists encourage clients to continue working, it is all about activity and who performs it.

### 3.3 Personal address

In personal address, four themes were identified: Welcome In, Let Me Help You, You Affect Me, and I Am Human.

Statements included in this category may also be examples of therapists affirming or encouraging clients. However, the coding of this category focused on the part of the sentence with some form of personal address. For example, a sentence such as “I'm glad to hear you've done the exercise” is a statement that would be considered both encouragement (for working with the treatment) and personal address (by addressing the therapist's feelings).

#### 3.3.1 Welcome In

In this theme, therapists invite clients to the treatment and a fellowship with the therapist. This includes unconditional care for the client, not contingent on counter-performance or effort.

*If you have any questions, just send me a message via the platform, I'm here!*

(T4)

The therapists look at clients and their suffering without judging, appraising, interpreting, or expecting anything.

*I can promise you that there is nothing you can tell me that I will think is strange or wrong.*

(T2)

The therapists are also clear on the fact that clients are no longer alone: the therapists believe in the clients, and the treatment, and hope that clients will feel better, no matter what the clients are thinking right now.

*I really want you to get the most out of this treatment*

(T5)

#### 3.3.2 Let Me Help You

This theme includes caring about clients and clients are invited to a “we” – therapist and client – who will go through treatment together. This differs from Welcome In as the therapists want clients to offer something in return – their stories, thoughts, and feelings. Here, therapists show interest in the clients, ask them to talk, and present themselves as ready to accept whatever the clients are about to tell them.

*I'm thinking we will work together to change your well-being for the better based on the situation you are in right now.*

(T4)

*Looking forward to working with you!*

(T3)

Therapists often motivate their requests for clients' stories by saying it will help the therapists help the clients. Storytelling is a way into cooperation. This distinguishes this instruction from the more unconditional storytelling of Welcome In. This way, this theme, despite its gentleness, becomes more focused on treatment and outcome.

*The more I know, the better I will understand your problems and help you with them!*

(T1)

#### 3.3.3 You Affect Me

Here, therapists use themselves by saying that the clients' actions and stories stir up emotion in the therapists. Therapists are happy when clients complete exercises, worried when clients do dangerous things, and curious before a chat. This theme is about therapists emphasizing the emotional bond between clients and therapists, and that the clients can affect the therapists.

*I've read what you've written for week two and the beginning of week three. Your list of what you do too much and too little, and I am once again deeply moved by what you write.*

(T1)

*Great to see that you completed both chapter five and chapter six exercises! It made me so happy to see that.*

(T3)

#### 3.3.4 I Am Human, too

This theme includes descriptions of how therapists are more than just any practitioners. This includes statements with personal information more detailed than expressing emotion to something the clients did or said. However, there is a similarity to You Affect Me, in that I Am Human, too is used by therapists as a reaction to something that the clients have said, in line with the instruction in Let Me Help You.

Within the category of personal address, this theme is the most personal in the sense that the therapists reveal things about themselves. This could be their own experiences of what clients are talking about and how the therapists felt or experienced that type of situation at the time.

*From my perspective, it is understandable that you are experiencing a lot of emotions at the same time. My parents are also divorced and I know it's really hard when they meet new partners.*

(T2)

It can be about the therapists' preferences.

*One trick that you can use when it comes to self-esteem, which I find usually helps, is to try to become your own best friend.*

(T5)

Another part of I Am Human, too, is therapists expressing how the treatment has been an important experience for them, too, and something they will remember. The therapists show their appreciation, and that the treatment has been of value to them.

*Thank you so much for letting me come along with you on this journey! It has been really rewarding for me to be part of your work with the treatment and to have gotten to see your progress! :)*

(T5)

## 4 Discussion

The purpose of this study was to explore written communication between internet therapists and clients, focusing on encouragement, affirmation, and personal address. Thematic analysis began with deductive coding based on the areas in focus. In this stage, 68 % of the content was extracted. This is in line with previous research, showing that supportive comments in general account for most of therapists' communication (Holländare et al., 2016; Paxling et al., 2013; Sánchez-Ortiz et al., 2011; Schneider et al., 2016).

Subsequent inductive coding showed that therapists encourage clients' hard work in treatment, portraying them as superheroes, and emphasizing their responsibility and understanding. Therapists affirm by saying that clients are having a hard time, that clients are like others, that it is time for a change, and by offering their views on the client's situation. Personal address appears when therapists invite clients to treatment and fellowship, and when therapists ask clients to tell their stories. Therapists also share their preferences and experiences and demonstrate that the treatment has been valuable to them, too.

### 4.1 Treatment as hard work

Describing treatment as hard work is central in the material, falling within the theme of Persistence Wins. It is also found in You Make Your Luck, where clients' responsibility for the work is emphasized. This idea of treatment also appears in other themes, for example in the encouraging You Are a Superhero, where the client who will perform the hard work is created, and in the affirming Hard Times, where the context of the hard work is described. Even in the personal address of Let Me Help You, the concept of hard work appears when the therapists offer a helping hand.

This overall pattern can be understood as therapists needing to convey that active effort from the client is required for depression to pass. As the current treatment was based on behavioral activation (BA), this is expected. The fact that clients must put in work between sessions is central in CBT (Tolin, 2016), as is the fact that changes in behavior lead to improved mental health and that you make changes one step at a time (Martell et al., 2010).

Because treatment is described as hard work and the client is expected to do the work, it is reasonable for therapists to encourage clients by telling them they have done a good job. This type of praise is a big part of Persistence Wins. One interpretation of why therapists frequently encourage clients in this way is that it is assumed to reinforce clients. An important task for therapists in BA is to encourage clients, and part of that can be praise. However, there are also clear instructions for therapists to focus reinforcement on problem-solving and what clients can learn from tasks, rather than results (Martell et al., 2010). In the current material, that kind of encouragement was not very prominent. Often, tasks that clients have completed are described in broader terms, followed by unspecific praise. The curious questions, described as signs of good reinforcement in BA, by Martell et al. (2010) are often missing. And here, this may be one of the elements in which face-to-face and online therapy differ, it is not easy to build up a two-sided communication with questions and answers if you write and read during different times, or only have small chats that perhaps do not evoke deeper conversations for which people would have to write long essay-like text.

Also common were therapists writing about their feelings regarding the clients' behavior. In this study, that falls under personal address and appears in You Affect Me. This is similar to encouraging comments, as the therapists are the ones who, through their reaction, indicate whether clients have done a good job. The idea could be to motivate clients to put in work, through them knowing that what they do (or do not do) matters to the therapists. Using therapists' feelings thus becomes another way for them to reinforce and build commitment and alliance. A way to overcome the gap that is shaped by the distance in room and time.

Putting too much trust in therapists' feelings and praise acting as reinforcers for clients could present a risk. If a client's main incentive through treatment has been to please or get praise from the therapist, this may affect motivation as treatment comes to an end. Tolin (2016) stresses the importance of avoiding the client becoming dependent on reinforcement from the therapist.

The great advantage of natural reinforcers is that they have a better chance of lasting, compared to arbitrary reinforcers (Martell et al., 2010). The therapists in this study are temporary visitors in the clients' lives, and the reinforcement from therapists will cease. Another potential disadvantage of reinforcement using therapists' feelings is that it is unknown whether it is reinforcing in all cases (Martell et al., 2010). Thus, natural reinforcers are more reliable, albeit a greater challenge not least in ICBT, where knowledge of clients' lives is often limited compared to face-to-face treatment preceded by a thorough assessment.

While therapists need not refrain from mentioning their feelings in the ways described in You Affect Me, if it would occur at the expense of guiding clients to natural reinforcers in everyday life, the treatment may not be as effective. Similarly, the praise that is a big part of Persistence Wins certainly has its place, but there may be a limited number of times messages such as “Good job!” maintain a reinforcing effect. Qualitative research has shown that large doses of therapist support can be perceived as aversive as it makes clients doubt their capability (Bendelin et al., 2011). A peppering of praise could share that effect and thus affect treatment negatively. Praise may also take up space from the recommended form of encouragement in BA: taking a curious stance and reinforcing clients' problem-solving ability.

### 4.2 Self-disclosure is rare

You Affect Me is a major theme within personal address. Something that also occurs, albeit rarely, is self-disclosure, confirming previous research (Holländare et al., 2016). Examples of self-disclosure are found in I Am Human. One explanation for the rare occurrence could be that the therapists are unaccustomed to being personal. Lacking knowledge of what characterizes appropriate and inappropriate self-disclosure (Hadjistavropoulos et al., 2018), therapists may choose not to reveal anything about themselves and use other techniques to follow the instruction of being personal.

### 4.3 Therapy for active superheroes

In the recommendations to the therapists, validation and normalization are important parts, just as in BA. Research on therapist behavior also shows that this is something that internet therapists engage in. In our study, validation and normalization occur mainly in the affirmation themes Hard Times and You Are Like Others.

When the therapists mention traits that may be associated with weakness, it is usually as an affirmation in Hard Times and You Are Like Others, where therapists validate clients and normalize suffering. In connection with validation, or as the next step, the weakness that was first validated is often contrasted or toned down. One example is when therapists distinguish between the clients and the depression in You Are Like Others. Another example is when the therapists go from validating clients to emphasizing how the fact that clients have sought help is a sign of strength, as described in Time for a Change.

Strength is also important in the encouragement theme of You Are a Superhero, which ties into the hard work that is a dominant pattern in the material. The therapists consistently paint a picture of clients as strong superheroes who will get through treatment. This can be seen as therapists wanting to show that they believe in the clients. However, it may also convey the idea that treatment is only for, or only helpful for, those who are strong, awesome, and brave.

The therapists further generate the idea of strong clients by interpreting activity as a sign of or synonymous with strength. A possible alternative would have been to consider weakness neither a prerequisite for nor an obstacle to working in treatment, thus arguing: “I know you are weak, but you can still put in work”. The idea of the superhero theme is probably to support the clients in treatment, but it is possible that this theme, with its intensity, may topple rather than help clients. There is a risk that depressed clients feel more like wrung-out dishcloths than superheroes, not recognizing the image that therapists are painting. Encouragement conveying that even wrung-out dishcloths can do CBT could potentially have offered a broader picture of who is capable of undertaking treatment. When encouragement focuses on results, as previously discussed, strength becomes the means to an end. Had therapists spoken more of the fact that problem-solving drives change, clients' weaker sides could have been framed as learning opportunities.

### 4.4 Therapists may choose the manual or a path of their own

The theoretical basis of the treatment is clear in My View on the Matter. Here, the therapists explain situations that have been normalized and validated in Hard Times and You Are Like Others. The explanation is CBT. In clients' messages, treatment components are prominent, with a strong focus on behavioral activation, but also cognitive restructuring, anxiety management, and exposure. The therapist creates a kind of an affirmation chain, where they validate and normalize and then, based on the clients' situation and in a personalized manner, present how CBT could help them understand and change their situation. Thus, therapists, especially in affirmation, actively try to tailor treatment to individual clients. The therapist support allows for individualization and a deeper understanding of CBT, based on the clients' situation.

### 4.5 Unique to the internet

An important question is to what extent the current findings are influenced by the fact that the treatment was internet-based. Instructions to therapists in BA are written for face-to-face treatment. Therapists' praise and talk of their feelings have been interpreted as ways to reinforce clients' work in treatment. However, there may be other reasons as to why these themes are so dominant. The therapists' choice to discuss their feelings can be a way of signaling that there is a human being on the other side of the screen, rather than a robot. “It made me happy when you completed the task” can be a therapist's way of compensating for the fact that they cannot smile or otherwise physically display interest. If so, this can be viewed as a supportive comment and a way for the therapist to build alliance (Sánchez-Ortiz et al., 2011). With the phrase “Good job!”, perhaps therapists simply want to communicate that they have seen an exercise or read a message, as they are unable to show it in any other way. The internet context may also explain the lack of curious questions that usually characterize good encouragement in BA. In this case, therapists are instructed not to encourage clients to respond to messages, but to refer questions to the chat. Perhaps this leads to the therapists avoiding curious questions in messages, as that could prompt a response from the client.

Another theme where the internet context should be considered is You Understand. The prominence of understanding was surprising, as understanding is not usually described as key in CBT. In CBT, insight is not considered enough for change, although some researchers may portray it as one step along the way (Grosse Holtforth et al., 2007). A possible explanation for therapists stressing clients' understanding is that they do not offer their rationale, an important ingredient in face-to-face CBT. Also, there is no room for individualized case formulation. In a rationale, therapists describe their view on how to tackle clients' problems based on individualized case formulations. The clients acceptance of the rationale affects whether they are prepared to subject themselves to demanding parts of the treatment (Tolin, 2016). It is possible that understanding has such a prominent place in the current content because the therapists are trying to compensate for the lack of rationale and individualized case formulation.

### 4.6 Method discussion

#### 4.6.1 The limitations of the concepts

Encouragement, affirmation, and self-disclosure, as defined by Holländare et al. (2016), were used as the basis for the analysis. However, the definitions of encouragement and affirmation are not obvious. For example, encouragement can be seen as an expression of affirmation, and the phrase ‘Good job!’ can be seen as praise rather than encouragement. All researchers studying therapist behavior must draw lines between concepts without clear boundaries. As was done in this paper. The current choices were based on the intention to draw conclusions in relation to previous studies.

Affirmation was defined as validating, interpreting, normalizing, and summarizing. In the findings, there are patterns of validation (Hard Times), normalization (You Are Like Others), and interpretation (My View on the Matter). This could be seen as circular reasoning: focus on validation, and you will see validation. These behaviors could also have had a different content, and so the themes show the content of, for example, validation, not validation itself. Validation was also found in other themes.

The selection of data and the analytical approach also need to be considered to evaluate the trustworthiness of this research. A limitation may be seen in the choice of a limited number of participants, but the accounts they gave were many in number, rich and detailed. Also, the use of documents instead of interview material promotes the credibility of the findings that depict more authentically what therapists really do, instead of what they are able to verbalize and remember in interview situations. The disadvantage of document analysis – not allowing for follow-up questions to secure correct understanding - was compensated by the fact that the authors who did the data analysis themselves had experiences of giving online therapy. This has helped to contextualize and interpret the material as well as it has motivated the choice to limit the analysis to semantic content instead of higher levels of abstraction and meaning-making. Further steps to secure that analysis and interpretation were grounded in the data and not driven by the authors' preconceptions and own experiences were taken to enhance the quality of this research. In more detail, all analytic work was conducted with rigor and in a systematic way, e.g., by writing an analytical protocol, and presenting and discussing analytical steps and possible interpretations in the author team High level of transparency was aimed for in the research report to improve confirmability.

### 4.7 Proposed future research

Research on internet-based psychological treatment was initially based on face-to-face treatments transferred to an online format. As research into psychotherapy via the internet has expanded, the trend has started to turn (Andersson, 2018). Now, novel ICBT interventions have been transferred to the face-to-face format (e.g., Rozental et al., 2018). Studies like the current can similarly improve not only knowledge on ICBT, but face-to-face treatment as well. Internet-based psychotherapy could be an engine in the development of psychotherapy in general, as it is less expensive, more accessible, and easier to monitor than face-to-face therapy. Qualitative research is important for this innovative engine as it can provide an in-depth picture and inform quantitative research.

This study did not provide any guidance regarding does-response. Future studies should actively manipulate the frequency, volume, and content of therapist responses. More research is needed on specific therapist behavior that seems effective, such as self-disclosure which appears to be important, yet uncommon. More detailed and experimental research on effective ways for therapists to use themselves is needed. In addition, comparing and contrasting therapist behaviors in ICBT to IPDT, focusing on similarities and differences, could be valuable for the understanding of possible common factors.

As asynchronous communication in the present study was complemented by synchronous communication, e.g., chats, in internet-based psychotherapy, knowledge of conversation in such context must be acquired and expanded. There are questions about the interaction between client and therapist, and what room for maneuvering there is in ICBT. Qualitative methods for exploring language use and interaction, such as discourse psychology (Lester et al., 2018) and conversation analysis (Paulus et al., 2016), could be appropriate. Analyses of how content is affected through the therapy process would also be valuable.

In previous research, encouragement, affirmation, and self-disclosure from therapists have been linked to outcomes in ICBT. This paper shows how these behaviors, and other types of personal address, manifest in treatment for young people with depression, and how that may be understood. We have shown how therapists can act, but what therapists should do remains to be explored. Future researchers will have to inform us of the best way of conveying ‘Good job!’.

## Declaration of competing interest

The authors declare that they have no known competing financial interests or personal relationships that could have appeared to influence the work reported in this paper.

## Appendix A Supplementary data

Appendix 1 Deductive code template. Appendix 1
