# Supplementary material for: “Good job!”: Therapists' encouragement, affirmation, and personal address in internet-based cognitive behavior therapy for adolescents with depression
Source: Internet Interv. 2022 Nov 15;30:100592. doi: 10.1016/j.invent.2022.100592 (PMC9682339; doi:10.1016/j.invent.2022.100592)
Supplement: Appendix 1 — Deductive code template. [file mmc1.docx]

**Appendix 1**

**Deductive Code Template**

Template for deductive and selective coding. The purpose of the coding was to extract the data set that the rest of the analysis would cover from the study's data corpus consisting of therapists' messages in five treatments. The template is primarily based on Holländare et al.'s (2016) definitions of therapist behaviours.

***Encouragement***

Therapist behaviors are aimed at encouraging some type of client behavior. This includes:

-  Reinforcing the client’s past behaviors. Examples: ‘Good that you noticed your feelings in that situation.’ ‘Thanks for your message!’

-  Encouraging statements about future behaviors that the client is planning, i.e., prompting future behaviors. Examples: ‘It's great that you are going out for coffee with a friend!’ ‘I look forward to reading your exercises.’

It includes behaviors expected in CBT treatment, such as exercises and other desirable behaviors that are good for the client.

-  Encouraging behaviors through cheerful and peppy exclamations. Examples: ‘Good job!’, ‘Super!’, ‘Great!’.

***Affirmation***

Pay attention to, acknowledge, and express interest in the client's thoughts, feelings, and actions and consider them valid. This includes:

-  Validating and interpreting: affirm by interpreting and validating what the client writes. Examples: ‘That must be hard for you’, ‘You are absolutely right in that...’. This also includes clarifying questions that help interpret and test hypotheses. Example: ‘Does that mean you don't feel so depressed anymore?’

-  Normalising: Affirm by writing that the client's behaviors are expected. ‘It's common to isolate yourself when you feel bad.’ ‘Panic is unpleasant, but it's not dangerous.’

-  Summarising: Affirm by summarising and repeating what the client wrote. Examples: ‘So you listen to audiobooks when you do your exercises.’ ‘You write that...’, but also ‘Today we talked about...’

***Personal address***

-  Self-disclosure. Therapists mention their own experiences and use personal examples from their own lives. Examples: ‘I also get bored of working out’, ‘I use this exercise myself when...’

-  Speaking in the first person and referring back to yourself. Examples: ‘What you write makes me wonder if...’ ‘You cannot disappoint me.’

-  Using yourself as a natural reinforcer. Examples: ‘It would make me so happy if you did the exercise.’ ‘It makes me happy when...’

-  Making yourself visible as a person.
